# Supplementary material for: Implication of p16 Promoter Methylation, the BRAFV600E Mutation, and ETS1 Expression Determination on Papillary Thyroid Carcinoma Prognosis and High-Risk Patients’ Selection
Source: Biomedicines. 2025 Jun 27;13(7):1583. doi: 10.3390/biomedicines13071583 (PMC12292734; doi:10.3390/biomedicines13071583)
Supplement: Supplementary file 1 [file biomedicines-13-01583-s001.zip › biomedicines-3702383-supplementary.pdf]

Supplementary Table S1: Association of *p16* promoter methylation with the occurrence of unfavorable clinicopathological parameters of PTC patients.

Clinicopathological Parameter of PTC		*p16* Methylated (n)		*p*-Value
		no	yes	
Gender	female	18	23	0.660
	male	6	10	
Intraglandular dissemination	no	11	14	0.798
	yes	13	19	
Lymph node metastasis	no	20	26	0.668
	yes	4	7	
Extrathyroid invasion	no	18	22	0.497
	yes	6	11	
Degree of tumor infiltration	1	8	8	0.552
	2	8	8	
	3	2	6	
	4	6	11	
pT	T1	4	6	0.601
	T2	12	13	
	T3	8	12	
	T4	0	2	
pTNM	I	9	10	0.861
	II	7	10	
	III	6	8	
	IV	2	5	

PTC: Papillary thyroid carcinoma, n: number of cases. p-value: statistical significance tested by χ^2-test.
